# Neuronal Networks during Burst Suppression as Revealed by Source Analysis

**DOI:** 10.1371/journal.pone.0123807

**Published:** 2015-04-30

**Authors:** Natia Japaridze, Muthuraman Muthuraman, Christine Reinicke, Friederike Moeller, Abdul Rauf Anwar, Kidist Gebremariam Mideksa, Ronit Pressler, Günther Deuschl, Ulrich Stephani, Michael Siniatchkin

**Affiliations:** 1 Department of Neuropediatrics, Christian-Albrechts-University, Kiel, Germany; 2 Department of Neurology, Christian-Albrechts-University, Kiel, Germany; 3 Department of Neurophysiology, Great Ormond Street Hospital for Children, London, United Kingdom; 4 Institute of Medical Psychology and Medical Sociology, Christian-Albrechts-University of Kiel, Kiel, Germany; University of British Columbia, CANADA

## Abstract

**Introduction:**

Burst-suppression (BS) is an electroencephalography (EEG) pattern consisting of alternant periods of slow waves of high amplitude (burst) and periods of so called flat EEG (suppression). It is generally associated with coma of various etiologies (hypoxia, drug-related intoxication, hypothermia, and childhood encephalopathies, but also anesthesia). Animal studies suggest that both the cortex and the thalamus are involved in the generation of BS. However, very little is known about mechanisms of BS in humans. The aim of this study was to identify the neuronal network underlying both burst and suppression phases using source reconstruction and analysis of functional and effective connectivity in EEG.

**Material/Methods:**

Dynamic imaging of coherent sources (DICS) was applied to EEG segments of 13 neonates and infants with burst and suppression EEG pattern. The brain area with the strongest power in the analyzed frequency (1–4 Hz) range was defined as the reference region. DICS was used to compute the coherence between this reference region and the entire brain. The renormalized partial directed coherence (RPDC) was used to describe the informational flow between the identified sources.

**Results/Conclusion:**

Delta activity during the burst phases was associated with coherent sources in the thalamus and brainstem as well as bilateral sources in cortical regions mainly frontal and parietal, whereas suppression phases were associated with coherent sources only in cortical regions. Results of the RPDC analyses showed an upwards informational flow from the brainstem towards the thalamus and from the thalamus to cortical regions, which was absent during the suppression phases. These findings may support the theory that a “cortical deafferentiation” between the cortex and sub-cortical structures exists especially in suppression phases compared to burst phases in burst suppression EEGs. Such a deafferentiation may play a role in the poor neurological outcome of children with these encephalopathies.

## Introduction

Burst suppression (BS) is an electroencephalogram (EEG) pattern characterized by the pseudo periodic alternant phases of high voltage activity (burst, 150–350 μV amplitude) and electrical silence (suppression, less than 25 μV amplitude), which is considered as a global state of profound brain inactivation [1]. Burst suppression can occur during different conditions: Early-Onset Epileptic Encephalopathies such as Ohtahara Syndrome and Early Myoclonic Encephalopathy [2], coma [1,3], hypothermia [4], and general anesthesia [5]. That all these different conditions produce similar brain activity may suggest that there is a unifying pathophysiological mechanism underlying this broad range of inactivated brain states. While the mechanisms of BS are still poorly understood, different etiologies resulting in BS indicate that the BS pattern represents a low-order dynamic mechanism that persists in the absence of higher-level brain activity [6]. This further infers that there may be a common pathway leading to the state of brain inactivation which may indicate a change in the fundamental properties of the brain’s arousal circuits [7].

There are only a few experimental studies trying to understand mechanisms of BS. In vivo studies of anaesthetized cats, which aimed to identify the potential cellular correlates of burst suppression, showed that during EEG flattening, up to 70% of thalamic cells were completely silent while the remainder showed rhythmic discharges in delta frequencies. Note, the deeper the burst suppression, the more thalamic cells completely ceased firing [8]. These findings were supported by a recent positron emission tomography (PET) study of an infant with an early myoclonic encephalopathy showing a profound hypoperfusion and hypometabolism of the basal ganglia and thalamus as well as cerebral cortex in the interictal period [9]. In contrast to interictal findings, an ictal SPECT investigation of the same patient revealed a significant hyper-perfusion of the bilateral basal ganglia, thalamus, brainstem, and deep cortical layers of bilateral fronto-parietal cortices suggesting a functional deafferentation of the cortex from subcortical structures in early myoclonic encephalopathy [9]. In summary, previous studies have shown the thalamus, basal ganglia, brainstem and especially the fronto-parietal cortex, are all involved in the generation of BS. However, it remains unclear, which structure or structures are responsible for bursts, suppression, and further, how the temporal dynamics between these structures may explain the alternating pattern of burst and suppression.

Due to a poor temporal resolution, it remains difficult to use either PET, SPECT or functional magnetic resonance imaging (fMRI) in order to answer these questions. Thanks to higher temporal resolution (millisecond range) EEG provides a better option for the analysis of neuronal networks underlying short transient events such as phases of bursts and suppression [10,11,12,13,14]. However, scalp EEG is spatially blurred due to the ambiguity of the underlying static electromagnetic inverse problem [15]. Furthermore, it is particularly difficult to carry out electrical source imaging of deep brain structures [16,17,18].

Recent developments in EEG inverse solutions have substantially improved the localization efficiency of EEG. This has enabled the use of EEG data for investigations into neuronal networks, even within deep structures in the brain [19,20,21]. One such method, Dynamic imaging of coherent sources (DICS), allows detection of brain regions that are either coherent with each other, coherent with a reference signal or coherent with a brain region [22]. It operates in the frequency domain for EEG and magnetoencephalogram (MEG) data by employing a spatial filter, and it is able to describe neuronal networks by both imaging power and coherence of oscillatory brain activity [22]. Applied to different types of tremor and epilepsies, DICS was able to characterize networks including thalamus, cerebellum and brainstem in MEG studies [22,23,24,25,26,27] as well as the thalamus and brainstem in recent EEG studies [19,20,21,28,29]. However, DICS cannot describe interaction between the sources [21,23,30,31,32,33,34,35]. In order to analyze effective connectivity i.e. information flow between sources, renormalized partial directed coherence (RPDC) can be used [13,36,37].

Here, we examine the neuronal networks underlying burst and suppression phases in neonates and infants with severe encephalopathies using electrical source imaging in order to identify common pathways to the state of brain inactivation, which may contribute to a better understanding of fundamental properties of the brain’s arousal circuits [6]

## Subjects and Methods

### Subjects

**Fig 1:**
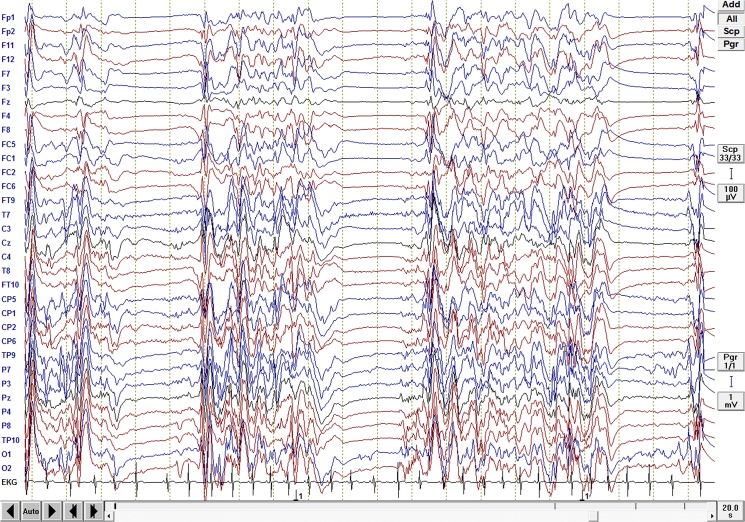
Burst Suppression EEG. BS-EEG from a patient with EME, showing alternative periods of slow waves of high amplitude (the burst) and periods of so called flat EEG (the suppression).

Thirteen infants and neonates with severe epileptic and non-epileptic encephalopathies with BS-EEG pattern were selected for the study. Four patients were recruited from the database of the Department of Neuropediatrics at the University Hospital of Schleswig-Holstein, campus Kiel and the Northern German Epilepsy Centre for children & adolescents, Schwentinental/Raisdorf, Germany. Nine patients were selected from the Department of Neurophysiology, Great Ormond Street Hospital for Children NHS Foundation Trust, London, UK. Clinical and demographical data of the patients are presented in Table 1 (age ranged from one day to one year old, mean age 4.3 months). All patients had global developmental delay of various severity which was assessed by neurological examinations. The majority of the patients had intractable seizures (six patients had no history of clinical seizures to date) (see Table 1). All of the patients had BS EEG pattern (see Fig 1). Four patients had severe hypoxic ischemic encephalopathy, six patients had epileptic encephalopathies, and three patients had neurometabolic disorders. Diagnoses were made according to the ILAE 2001 classification scheme (Commission on Classification and Terminology of the International League against Epilepsy, 2001). The neurological examination and structural MRI were performed in all cases. Routine EEGs (in accordance with the 10–20 system) were recorded in all cases and were independently evaluated by two neurophysiologists who confirmed the type of EEG abnormality.

**Table 1:** Demographic and clinical Data of the patients.

Pat.	Diagnose	MRI findings	Age at EEG	AED	Type of seizures
1^1^	HIE ^a^	Signal abnormality and swelling of basal ganglia and thalami	1 day	PB	No clinical seizures
2^1^	HIE ^b^	Global cerebral, basal ganglia and thalamus infarction	13 mo	MDL	No clinical seizures
3^1^	Epileptic encephalopathy	brain malformation, simplified gyration, hypoplastic cerebellum	1 mo	none	No clinical seizures
4^1^	Neurometabolic disease ^c^	Multiple, bilateral hemorrhages	5 day	none	No clinical Seizures
5^1^	HIE, IC hemorrhage	Brain atrophy ^d^	3 days	PB	No clinical Seizures
6^1^	Migrating epilepsy	Normal	7 mo	PHT, CLZ, ZNS, Ketogenic diet	Focal motor and Tonic seizures
7^1^	Non-ketotic hyperglycinaemia	Mild delay of myelination	2 mo	PB	Tonic seizures
8^1^	HIE, ICH	Intracerebral Heamorrhage, oedema^e^	6 days	PB, PHT	No clinical seizures
9^1^	Metabolic	bilateral perisylvian and insular polymicrogyria	6 weeks	PB	Tonic seizures
10^2^	Focal epilepsy. later West-Syndrome	Delay of myelination, potentially focal cotical dysplasia	8 mo	OXC, VPA, STP	Focal tonic seizures
11^2^	EME DD: Otahara	Delay of myelination, progressive atrophy of white matter	11 mo	PHT, CLB, TPM, CLB	Tonic seizures in series, polytopic myoclonia
12^2^	Focal epilepsy. later West-Syndrome	No definitely pathological finding	1 yr	STM, VGB	Tonic-clonic seizures
13^2^	Focal epilepsy. later West syndrome	Myelination delay, focal cortical dysplasia of the left frontal lobe	9 mo	PHT, CLB	Epileptic spasms in series

**List of abbreviation**: AED Antiepileptic drugs; EEG Electroencephalography; PB Phenobarbital; MDL Midazolam; PHT Phenytoin; CLZ Clonazepam; ZNS Zonisamid; OXC—Oxcarbazepin, CLB Clobazam, STP Stiripenton. VGB Vigabatrin, STM Sulthiam. HIE hypoxic ischemic encephalopathy; IC intracranial Hemorrhage;

^1^ Patients from the Department of Neurophysiology, Great Ormond Street Hospital for children, London, UK.

^2^ Patients from the Department of Neuropediatrics at the University Hospital of Schleswig-Holstein, campus Kiel and the Northern German Epilepsy Centre for children & adolescents, Schwentinental/Raisdorf, Germany

^a^ Severe perinatal hypoxic ischemic encephalopathy

^b^ Severe hypoxic ischemic encephalopathy due to an aspiration with the grape and respiratory and cardiac arrest.

^c^ DD: thromboembolic disease or genetic / mitochondrial disease.

^d^ Marked brain atrophy, almost no remaining occipital parenchymal tissue. No underlying structural cause for the previous hemorrhage.

^e^ Intraventricular haemorrhage, hydrocephalus. Diffuse cerebral hemisphere oedema. Left cerebellar and infra and supratentorial subdural haematoma, A small non-compressive intradural haematoma in the upper thoracic spine, subarachnoid haemorrhage.

The study was acknowledged by the Ethics Committee of the Faculty of Medicine, University of Kiel, Germany and was conducted according to the Declaration of Helsinki (current version, 1996) on biomedical research involving human subjects (Tokyo amendment). The study was registered and approved by the research and development office of the UCL Institute of Child Health, London, United Kingdom. Parents or legal guardians of participants were informed about the research purposes and gave verbal informed consent, which was not recorded, to keep the procedure anonymously. This procedure was also approved by the Institutional Review Boards.

### EEG analysis

#### EEG recording

For the patients recruited from the Department of Neuropediatrics at the University Hospital Schleswig-Holstein and the Northern German Epilepsy Centre for children & adolescents standard EEG recordings were performed according to the 10/20 system (EEG recording system: Neurofile; IT-med, Bad Homburg, Germany). In some cases following additional electrodes were used for the analysis: FC1, FC2, FC5, FC6, CP1, CP2, CP5, CP6, FT9, FT10, TP9, TP10, ECG. Impedance was kept below 10 kOhms, Sampling rate was 512 Hz. Reference was located between Fz and Cz. If required, EEGs were further processed for the correction of ECG artifacts. All EEGs were recorded during sleep.

For the nine patients recruited from the Department of Neurology, Great Ormond Street Hospital for children, London standard EEG recordings, according to a modified 10/20 system (EEG recording system: Natus XLTek, Oakville, Ontario, Canada) were used for the analyses. Following parameters were used during the recording: impedance was kept below 10 kOhms, Sampling rate: 512 Hz. Reference was located between Cz and Pz. If required, EEGs were further processed for the correction of ECG artifacts. All EEGs were recorded during sleep.

#### Selection of EEG epochs

**Fig 2:**
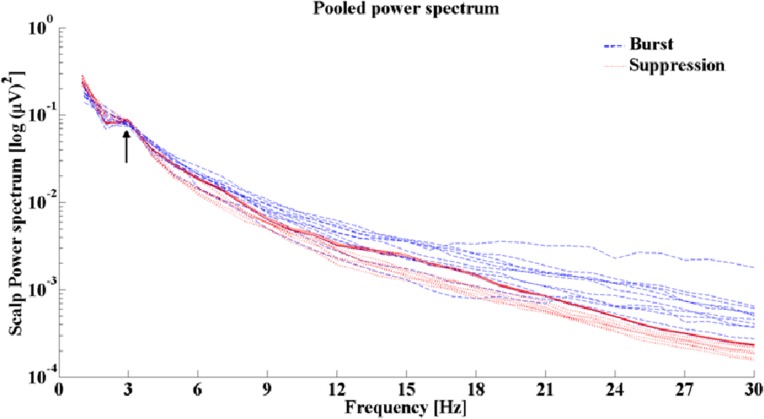
Pooled power spectrum. Pooled power spectrum showing a clear peak at the 1–4 Hz frequency range in all these patients for both the burst and suppression phases

The EEG segments were visually inspected by two experienced neurophysiologists independently. For an appropriate DICS analyses, long EEG segments are necessary in order to achieve an acceptable signal-to-noise ratio. Therefore, EEG segments with the burst phases were selected, marked and segmented, i.e., cut out from the entire EEG recording and then concatenated together to acquire EEG segments containing 60 seconds of burst-only phases. The same was performed for the suppression phases, so that eventually 60 seconds duration suppression phases were analyzed for each patient. Power spectrum analyses were performed to identify the predominant frequencies in both phases (Fig 2). The FFT analysis revealed the predominant frequency ranging from 1–4 Hz in all patients for both burst and suppression phases. This frequency range was used for further analyses.

#### Spectral Analysis

A multitaper method [38] was used to compute the power spectra for all recorded EEG channels separately for burst and suppression phases. The data epoch of one second length was tapered using a set of discrete prolate spheroidal sequences [39]. The Fourier-transformation was applied to the tapered data epochs and auto-spectra were computed. Subsequently, the spectra were averaged and the power spectrum was estimated. For more detailed description refer to [40]. The main frequency band was then defined accordingly for the subsequent source analysis. The burst and suppression phases have different amplitudes in the raw EEG so in order to evaluate the implications of this change in amplitude on the source analysis, the relative signal to noise ratio (SNR) was estimated by calculating the power at the frequency band 1–4 Hz for the burst as the numerator and the suppression phase as the denominator in each patient. The relative SNR in all the patients ranged from (35.85–39.09 dB). In a further analysis, all initial power estimates of the individual EEG electrodes were combined to get a pooled power estimate. This can be done by computing the individual second order spectra using a weighting scheme and estimate the power to obtain the pooled estimate as previously described [41,42].

#### Source Analysis

DICS [22] was used to find the sources in the brain responsible for the delta activity during burst and suppression phases. Forward and inverse problems needed to be solved in order to locate the origin of specific EEG activity seen on the scalp. The forward solution was estimated with specified models for the brain. The brain was modeled by a more complex, five-concentric-spheres model [43,44] with a single sphere for each layer corresponding to the white matter, grey matter, cerebral spinal fluid, skull and skin. Standard T1 infant-neonate images (UNC School of medicine) [45] were used to construct the volume conductor model. A part of the forward modeling and the source analysis was done using the open source software Fieldtrip [46]. The lead field matrix was estimated as the next step which contains the information about the geometry and conductivity of the model. The complete description of the forward solution has been previously described elsewhere [47]. In order to determine the coherence between brain areas, the spatial maximum of the power was identified at the frequency band (1–4 Hz), and then defined as the reference region. The selection of the reference region and the subsequent coherent sources was done on an automated basis. The final step was to apply a spatial filter to estimate the source signals from these identified regions to study directionality [48]. For more detailed description of the source analysis please see our previous publications [19,20,21,28,29].

#### Directionality analysis

Coherent analysis does not provide information regarding the effective connectivity between the identified sources, it only describes sources that are coherent in a given frequency band. In order to study the direction of informational flow between each of the sources we applied the method of renormalized partial directed coherence (RPDC) [49] which is a technique performed in the frequency domain. The RPDC method uses a multivariate (MVAR) modeling approach to find the functional information flow between the bands of 1–4 Hz in our case. For more detailed description of the method see the following references [13,50,51,52,53,54]. The effective connectivity derived from EEG measurements is difficult to identify due to the presence of noise and the volume conduction effects [55]. In order to test the reliability of detecting the underlying neuronal interactions during any functional state of interest some authors used the imaginary part of coherence [56,57] or time reversal technique (TRT) [58]. In a recent simulation study, Haufe and colleagues [58] showed that the TRT is an appropriate method to improve the influence of weak asymmetries (i.e. non-casual interactions caused by zero-lagged coherences (= volume conduction) on the result of any causal measure, while maintaining or even amplifying the contribution of strong asymmetries (i.e. time-lagged causal interactions not caused by volume conduction). The bootstrapping method was used to calculate the significance level on the applied data followed by the TRT as a second significance test after the estimation of the RPDC values.

#### Statistical analysis

The significance of the sources was tested using a within-subject surrogate analysis. The surrogates were assessed using a Monte Carlo random permutation 100 times shuffling of one-second segments within each subject. The p-value for each of these 100 random permutations were determined, and then the 99th percentile p-value was taken as the significance level in each subject [59]. Next, for the statistical comparison on the source absolute power values, the mean source coherence (or interaction strength) and the RPDC values between all the sources were estimated for testing the significance between burst and suppression phases. A Friedman two-way analysis of variance test was then performed on the mean coherence values. We applied time reversal technique TRT as a second significance test on the connections already identified by RPDC using bootstrapping as a data-driven surrogate significance test. For all the statistical analyses, the significance level was kept at p < 0.01.

## Results

### DICS

#### Burst phase

**Fig 3:**
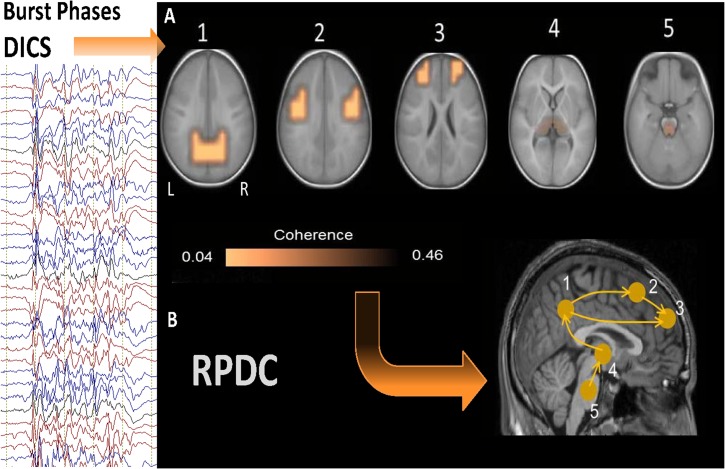
Grand avarage of DICS and RPDC results during burst phases. A. Grand average of the sources described by DICS analysis during burst phase. The source of the strongest power in the frequency band 1–4 Hz was detected bilaterally in precuneus in all 13 patients. The 2nd source was detected bilaterally in the somatosensory cortex in all patients. The 3rd source was detected in prefrontal regions bilaterally; Subsequent sources were detected in the thalamus (4th source) bilaterally and the last coherent source was found in the brainstem, in all 13 patients. B. RPDC during burst phase: showing significantly (p = 0.003) stronger information flow from the precuneus (source 1) towards the somatosensory cortex (p = 0.001) (second source) and the prefrontal cortex (third source) (p = 0.004) as well as from the brainstem (source 5) towards the thalamus (p = 0.004) and from the thalamus to the precuneus (p = 0.004) rather than vice versa. Also, stronger RPDC (p = 0.002) was detected from the somatosensory cortex towards the prefrontal cortex.

The grand average of the sources described by DICS analysis is shown in Fig 3 (results of DICS analyses for individual patients are shown in S1–S4 Figs). All these identified sources were statistically significant (p = 0.006) according to Monte Carlo random permutation across all the subjects. During burst phases, the source of the strongest power in the frequency band 1–4 Hz was detected bilaterally in the precuneus (BA 39 and BA 7) in all 13 patients. The local maximum of this source varied across the patients (S1–S4 Figs). This first source was defined as the reference region for further coherence analysis between brain areas. In all the cases, there were four sources coherent with the first source, and there were only small differences across the patients with respect to the local maxima of the sources (S1–S4 Figs). Sources with the strongest coherence with the reference source were found bilaterally in the somatosensory cortex (second source; BA 2 and BA 3) in the patients. The next strongest source were detected in prefrontal regions bilaterally (BA 9); subsequent sources were found in the thalamus (BA 23) bilaterally in eleven patients and unilaterally on the left side in two patients, whereas the last coherent source was determined in the brainstem (BA 25), or more precisely in the mid-brain tegmentum in all thirteen patients.

#### Suppression Phase

**Fig 4:**
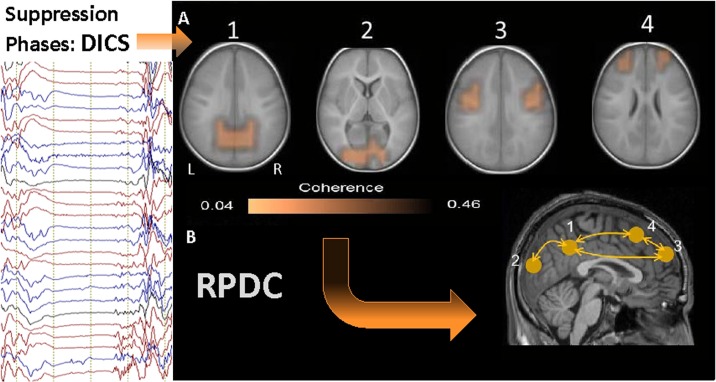
Grand avarage of DICS and RPDC results during suppression phases. A. Shows DICS results for the suppression phases. The source of the strongest power in the frequency band 1–4 Hz was detected bilaterally in the precuneus in all 13 patients (first source) and was very similar to the strongest sources during burst phases. The 2nd strongest sources were found bilaterally in the occipital cortex in 11 patients and unilaterally on the left side in two patients. The 3rd source was detected in the somatosensory cortex bilaterally. The last sources were detected bilaterally in the prefrontal cortex, similar to the third source during the burst phases. No deep sources were discovered during the suppression phases. B. RPDC during suppression phases showed no significant differences in information flow between sources. Connections between the sources: 1 and 2 (p = 0.49), 1 and 3(p = 0.52), 1 and 4 (p = 0.32), 2 and 3 (p = 0.29), 2 and 4 (p = 0.42), 3 and 4 (p = 0.29).

All the identified sources were statistically significant (p = 0.009) according to Monte Carlo random permutation across all the subjects. The source of the strongest power in the frequency band 1–4 Hz was detected bilaterally in the precuneus (BA 39 and BA 7) in all 13 patients (first source) and was very similar to the strongest source during burst phases (see Fig 4 and S1–S4 Figs). This strongest source was further used as the reference region for coherence analysis between brain areas. In all the cases there were three sources coherent with the first source, and there were only small differences across the patients with respect to the local coherence maxima of the sources (S1–S4 Figs). The second strongest source was found bilaterally in the occipital cortex (second source; BA 17) in eleven patients and unilaterally on the left side in two patients. This source was not present during burst phases. The next strongest coherence was detected bilaterally in the somatosensory cortex (third source; BA 2 and BA3), the location of these sources were analogous to the second sources during the burst phases. The last coherent source were determined bilaterally in prefrontal cortex (BA 9), similar to the third source during the burst phases. No sub-cortical sources were observed during the suppression phases.

### RPDC

#### Burst Phases

During burst phases RPDC showed that the direction of information flow was significantly stronger from the posterior regions towards the anterior regions. There was a significantly (p = 0.006) stronger information flow from the precuneus (source 1) towards the somatosensory cortex (p = 0.009) (second source) and the prefrontal cortex (third source) (p = 0.004). The upward information flow was from the brainstem (source 5) towards the thalamus (p = 0.003) and from the thalamus to the precuneus (p = 0.007) rather than vice versa. In addition stronger information flow (p = 0.002) was detected from the somatosensory cortex towards the prefrontal cortex (see Fig 3 and S5 Fig). All cortical regions showed a clear trend of significant information flow from posterior regions towards anterior regions. The other connections between sources 2 to 5 were not significant.

#### Suppression Phases

RPDC showed no significant differences in flow of information between the sources. This indicates bidirectional and homogenous informational flow (see Fig 4). However, all these bidirectional connections were significant in the data-driven bootstrapping method for the RPDC analyses (p > 0.1) (shown in S6 Fig) and TRT analyses revealed strong symmetries.

#### Comparison between burst and suppression phases

**Fig 5:**
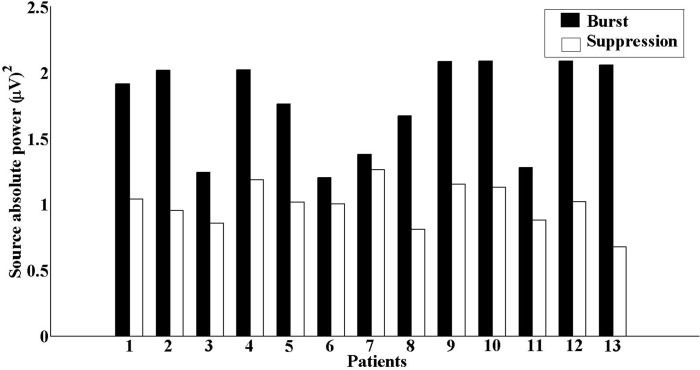
Source absolute power. The source absolute power for the first source during both phases showing higher absolute power during burst phases than during the suppression phases.

We compared the source absolute power between the two phases and found that the burst phases were significantly (p = 0.009) higher than those during the suppression phases (see Fig 5). Next, we compared the total interaction strength of coherence between the sources during both phases and found that coherence values during burst phases were significantly (p = 0.0006) higher than those during the suppression phases. In this study, the bootstrapping method followed by the TRT analyses underlined the robustness of the above-mentioned results, as any significant causal interaction identified by RDPC were identified as strong asymmetry by the TRT.

## Discussion

This study represents the first descriptive interpretation, by means of imaging power and coherence of oscillatory brain activities, of the dynamics of neuronal networks underlying the BS EEG pattern in neonates and infants with severe encephalopathies. Results of DICS analysis demonstrated that delta activity during the suppression phases was associated with coherent sources in precuneus, occipital cortex, somatosensory cortex and prefrontal cortex. However, DICS analysis during burst phases showed additional sources in thalamus and the brainstem. Furthermore, we demonstrated that burst phases were characterized by significantly higher source absolute power and mean coherence values between the sources and showed stronger informational flow between subcortical and cortical sources compared to the suppression phases. The significant difference in mean coherence is not due to the difference in power between the two phases, as shown via estimation of the relative SNR on the scalp level. The described network underlying burst and suppression phases was found on both group and individual levels. The networks represent a stable inter-individual pattern of functional and effective connectivity, which is independent of etiology, and represents a common mechanism of BS.

The strongest source during both the burst and suppression phases was in the precuneus/posterior cingulate cortex. These regions, together with the retrosplenial brain area, is known to be one of the critical nodes of neural network correlates of consciousness (NNCC) [60] and an important component of the default mode network [61,62]. Additionally, these regions have the highest level of brain glucose metabolism and cytochrome C oxidase activity [60]. It is known that metabolism in posterior cingulate and retrosplenial brain areas are likely to be driven by the projections from the thalamus. Furthermore, brainstem projections towards the thalamus provide a key coupling between the brainstem system for arousal, and cortical systems for cognitive processing and awareness [60]. The central thalamus receives upwards projections from the brainstem/basal forebrain “arousal systems” that control the activity of many cortical and thalamic neurons during the sleep-wake cycle [63]. These projections are present only during the burst phases, whereas during the suppression phases subcortical structures show no coherence/interaction with the cortical structures. These findings are in line with previous studies showing that up to 70% of thalamic cells are silent during the suppression phases [8]. Also, postmortem investigations of patients in vegetative state frequently showed damage to the mid-brain and thalamus [64,65]. Importantly, bilateral injuries of thalamus are also associated with global disorders of consciousness (coma, vegetative state, and minimally conscious state) [66,67]. Furthermore, PET and SPECT investigations of patients with early myoclonic encephalopathy (EME) suggested that there is a profound functional deafferentation of the cortex from subcortical structures, providing a new insight into the pathophysiology of BS pattern in EME [9]. These observation indicate that thalamic neurons play a causal role in disorders of consciousness [63].

During burst phases there are extensive projections from the basal-ganglia to the frontal lobe through the precuneus, whereas during suppression phases these projections are fully deorganized and show no informational flow towards frontal regions. Extensive and topographically organized basal-ganglia outputs to the prefrontal cortex, via the precuneus, influence cognitive operations of the frontal lobe [68]. Our findings support the theory that each burst phase can be seen as an attempt to recover normal neuronal dynamics, as proposed by Ching and colleagues [6]. By constructing a bio-physical computational model they showed that alternating features of BS may arise through interactions between neuronal dynamics and neurometabolism. They suggest that BS represents a basal neurometabolic regime that ensures basic cell function during states of lowered metabolism. Suppression occurs when there is an imbalance between neuronal activity and available energetics, whereas each successive recovery of rhythms within bursts is due to the recovery of the basal dynamics at the neuronal circuit level caused by the transient increases in energetics.

An alternate theory, proposed by Amzira and colleagues [69,70], further elaborates regarding the disruption of the excitatory–inhibitory balance. According to their theory, the burst phase is accompanied by an exhaustion of extracellular cortical Ca^2+^, which generates a general disconnection of cortical networks, subsequent arrest of neocortical neuronal activities, and the resulting flat EEG. However, during suppression phases, interstitial Ca^2+^ levels are restored to levels, whereby any external or intrinsic signal can trigger a new burst in the hyper-excitable cortex [69,70]. These findings coincide with those reported by Schiff and colleagues [71]. They proposed the existence of disorganized and “free running” corticostriatopallidal, thalamocortical and corticothalamic loops working at a very low metabolic level during chronic unconscious states.

**Methodological limitations:** In this study we reveal brain sources in sub-cortical regions such as the thalamus and brain stem. It has been a matter of debate for many years whether it is possible to find sub-cortical sources based on recordings on the scalp. In previous MEG [22,23,24,72] and EEG [59] studies, subcortical sources have been detected by applying DICS to oscillatory signals (e.g. tremor), and also in healthy subjects during isometric contraction [29]. This analytical method has also been shown to identify successfully subcortical sources in the thalamus [20,21] and the brainstem [19] in our previous studies based on EEG. There have been also two validation studies with an independent method like EEG-fMRI [20] and local field potentials measured simultaneously with EEG from macro electrodes in orthostatic tremor patients [47]. The second limitation is that we have not used realistic head modeling for the source analysis. Considering that current study is a retrospective study MRIs in cases of the investigated patients were not performed according to the requirements necessary for the optimal head modeling (3D T1 and T2 Images and DTI images). In addition, some of the sequences were not fully performed, i.e. parietal or temporal parts were incompletely scanned, which is a substantial obstacle for the head modeling. It is likely that the realistic head modeling would improve the localization power of DICS. The third limitation of our study can be that we have investigated suppression phases, i.e. phases with a very poor electrical activity of the brain, which is a challenge for the source analyses. Nevertheless we found the pooled power spectrum in both the burst and suppression phases showed clear peaks in the frequency range of 1–4Hz.

Furthermore, we investigated non-homogeneous group of patients, which were treated with different medications, investigated with different numbers of electrodes and at different time points. However, the aim of our study was to investigate neuronal networks in infants with BS pattern regardless of the etiology, severity of the disease or medications. The described network underlying burst and suppression phases was found at both group and individual levels. We therefore believe that it is as a fingerprint of the BS-EEG pattern, which, independent of etiology, represents a common mechanism of BS. This is in line with other studies which have shown that a certain EEG pattern, independent of etiological factors, can be represented by a common neuronal networks [12,19,20,73,74].

### Conclusion

In this study, we investigated dynamics within the neuronal networks during BS-EEG pattern in neonates and infants with severe epileptic and non-epileptic encephalopathies. Our study revealed that there is a specific periodicity of neuronal activity during BS. Each suppression phase shows a complete deafferentation between subcortical and cortical structures and cessation of neuronal projections responsible arousal and cognitive processing and awareness. However, each consecutive burst can be considered as a temporary recovery of subcortical and cortical neuronal dynamics. Despite the above mentioned limitations, our findings support the feasibility of the described methodology for the investigation of infants with severe encephalopathies with burst suppression EEG pattern.

## Supporting Information

S1 Fig DICS results for individual patients. DICS analysis results: showing all sources that were described by DICS in each patient separately for burst and suppression phases, numbered according to the strength of the identified sources. During burst phases: the source of the strongest power (source 1) in the frequency band 1–4 Hz was detected bilaterally in the precuneus in all 13 patients. The local maximum of this source varied across the patients. In all the cases, there were four sources coherent with the first source. Sources with the strongest coherence with the reference (source 2) source were found bilaterally in the somatosensory cortex in the patients. The next strongest source (source 3) were detected in prefrontal regions bilaterally; subsequent sources were found in the thalamus (source 4) bilaterally in eleven patients and unilaterally on the left side in two patients, whereas the last coherent source was determined in the brainstem (source 5), or more precisely in the mid-brain tegmentum in all thirteen patients. During suppression phases: The source of the strongest power (source 1) in the frequency band 1–4 Hz was detected bilaterally in the precuneus in all 13 patients. The second strongest sources were found bilaterally in the occipital cortex (source 2) in eleven patients and unilaterally on the left side in two patients. The next strongest coherence was detected bilaterally in the somatosensory cortex (source 3), the location of these sources were analogous to the second sources during the burst phases. The last coherent sources were determined bilaterally in prefrontal cortex (source 4).
(TIF)

S2 Fig DICS results for individual patients. DICS analysis results: showing all sources that were described by DICS in each patient separately for burst and suppression phases, numbered according to the strength of the identified sources. During burst phases: the source of the strongest power (source 1) in the frequency band 1–4 Hz was detected bilaterally in the precuneus in all 13 patients. The local maximum of this source varied across the patients. In all the cases, there were four sources coherent with the first source. Sources with the strongest coherence with the reference (source 2) source were found bilaterally in the somatosensory cortex in the patients. The next strongest source (source 3) were detected in prefrontal regions bilaterally; subsequent sources were found in the thalamus (source 4) bilaterally in eleven patients and unilaterally on the left side in two patients, whereas the last coherent source was determined in the brainstem (source 5), or more precisely in the mid-brain tegmentum in all thirteen patients. During suppression phases: The source of the strongest power (source 1) in the frequency band 1–4 Hz was detected bilaterally in the precuneus in all 13 patients. The second strongest sources were found bilaterally in the occipital cortex (source 2) in eleven patients and unilaterally on the left side in two patients. The next strongest coherence was detected bilaterally in the somatosensory cortex (source 3), the location of these sources were analogous to the second sources during the burst phases. The last coherent sources were determined bilaterally in prefrontal cortex (source 4).
(TIF)

S3 Fig DICS results for individual patients. DICS analysis results: showing all sources that were described by DICS in each patient separately for burst and suppression phases, numbered according to the strength of the identified sources. During burst phases: the source of the strongest power (source 1) in the frequency band 1–4 Hz was detected bilaterally in the precuneus in all 13 patients. The local maximum of this source varied across the patients. In all the cases, there were four sources coherent with the first source. Sources with the strongest coherence with the reference (source 2) source were found bilaterally in the somatosensory cortex in the patients. The next strongest source (source 3) were detected in prefrontal regions bilaterally; subsequent sources were found in the thalamus (source 4) bilaterally in eleven patients and unilaterally on the left side in two patients, whereas the last coherent source was determined in the brainstem (source 5), or more precisely in the mid-brain tegmentum in all thirteen patients. During suppression phases: The source of the strongest power (source 1) in the frequency band 1–4 Hz was detected bilaterally in the precuneus in all 13 patients. The second strongest sources were found bilaterally in the occipital cortex (source 2) in eleven patients and unilaterally on the left side in two patients. The next strongest coherence was detected bilaterally in the somatosensory cortex (source 3), the location of these sources were analogous to the second sources during the burst phases. The last coherent sources were determined bilaterally in prefrontal cortex (source 4).
(TIF)

S4 Fig DICS results for individual patients. DICS analysis results: showing all sources that were described by DICS in each patient separately for burst and suppression phases, numbered according to the strength of the identified sources. During burst phases: the source of the strongest power (source 1) in the frequency band 1–4 Hz was detected bilaterally in the precuneus in all 13 patients. The local maximum of this source varied across the patients. In all the cases, there were four sources coherent with the first source. Sources with the strongest coherence with the reference (source 2) source were found bilaterally in the somatosensory cortex in the patients. The next strongest source (source 3) were detected in prefrontal regions bilaterally; subsequent sources were found in the thalamus (source 4) bilaterally in eleven patients and unilaterally on the left side in two patients, whereas the last coherent source was determined in the brainstem (source 5), or more precisely in the mid-brain tegmentum in all thirteen patients. During suppression phases: The source of the strongest power (source 1) in the frequency band 1–4 Hz was detected bilaterally in the precuneus in all 13 patients. The second strongest sources were found bilaterally in the occipital cortex (source 2) in eleven patients and unilaterally on the left side in two patients. The next strongest coherence was detected bilaterally in the somatosensory cortex (source 3), the location of these sources were analogous to the second sources during the burst phases. The last coherent sources were determined bilaterally in prefrontal cortex (source 4).
(TIF)

S5 Fig Grand average of RPDC results for burst phases. Each bar in the graphs shows the mean and standard deviation of the strength of information flow between two given sources, separately for each direction. Dashed line illustrates the surrogate data driven significance level.
(TIF)

S6 Fig Grand average of RPDC results for suppression phases. Each bar in the graphs shows the mean and standard deviation of the strength of information flow between two given sources, separately for each direction. Dashed line illustrates the surrogate data driven significance level.
(TIF)
