# Supplementary figures and images for: Neuronal Networks during Burst Suppression as Revealed by Source Analysis
Source: PLoS One. 2015 Apr 30;10(4):e0123807. doi: 10.1371/journal.pone.0123807 (PMC4415810; doi:10.1371/journal.pone.0123807)

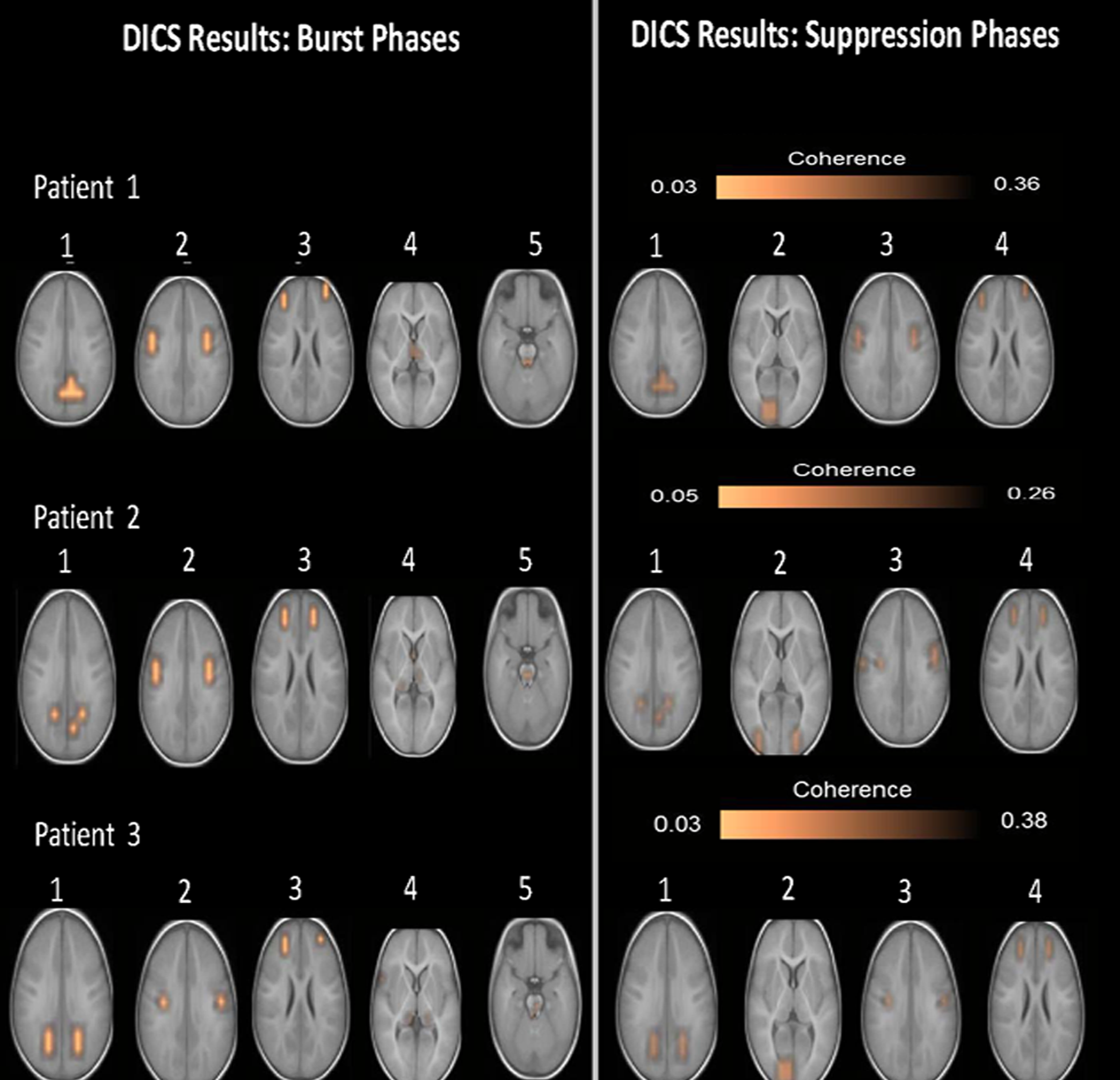

Supplement: S1 Fig — DICS analysis results: showing all sources that were described by DICS in each patient separately for burst and suppression phases, numbered according to the strength of the identified sources. During burst phases: the source of the strongest power (source 1) in the frequency band 1–4 Hz was detected bilaterally in the precuneus in all 13 patients. The local maximum of this source varied across the patients. In all the cases, there were four sources coherent with the first source. Sources with the strongest coherence with the reference (source 2) source were found bilaterally in the somatosensory cortex in the patients. The next strongest source (source 3) were detected in prefrontal regions bilaterally; subsequent sources were found in the thalamus (source 4) bilaterally in eleven patients and unilaterally on the left side in two patients, whereas the last coherent source was determined in the brainstem (source 5), or more precisely in the mid-brain tegmentum in all thirteen patients. During suppression phases: The source of the strongest power (source 1) in the frequency band 1–4 Hz was detected bilaterally in the precuneus in all 13 patients. The second strongest sources were found bilaterally in the occipital cortex (source 2) in eleven patients and unilaterally on the left side in two patients. The next strongest coherence was detected bilaterally in the somatosensory cortex (source 3), the location of these sources were analogous to the second sources during the burst phases. The last coherent sources were determined bilaterally in prefrontal cortex (source 4). (TIF) [file pone.0123807.s001.tif]

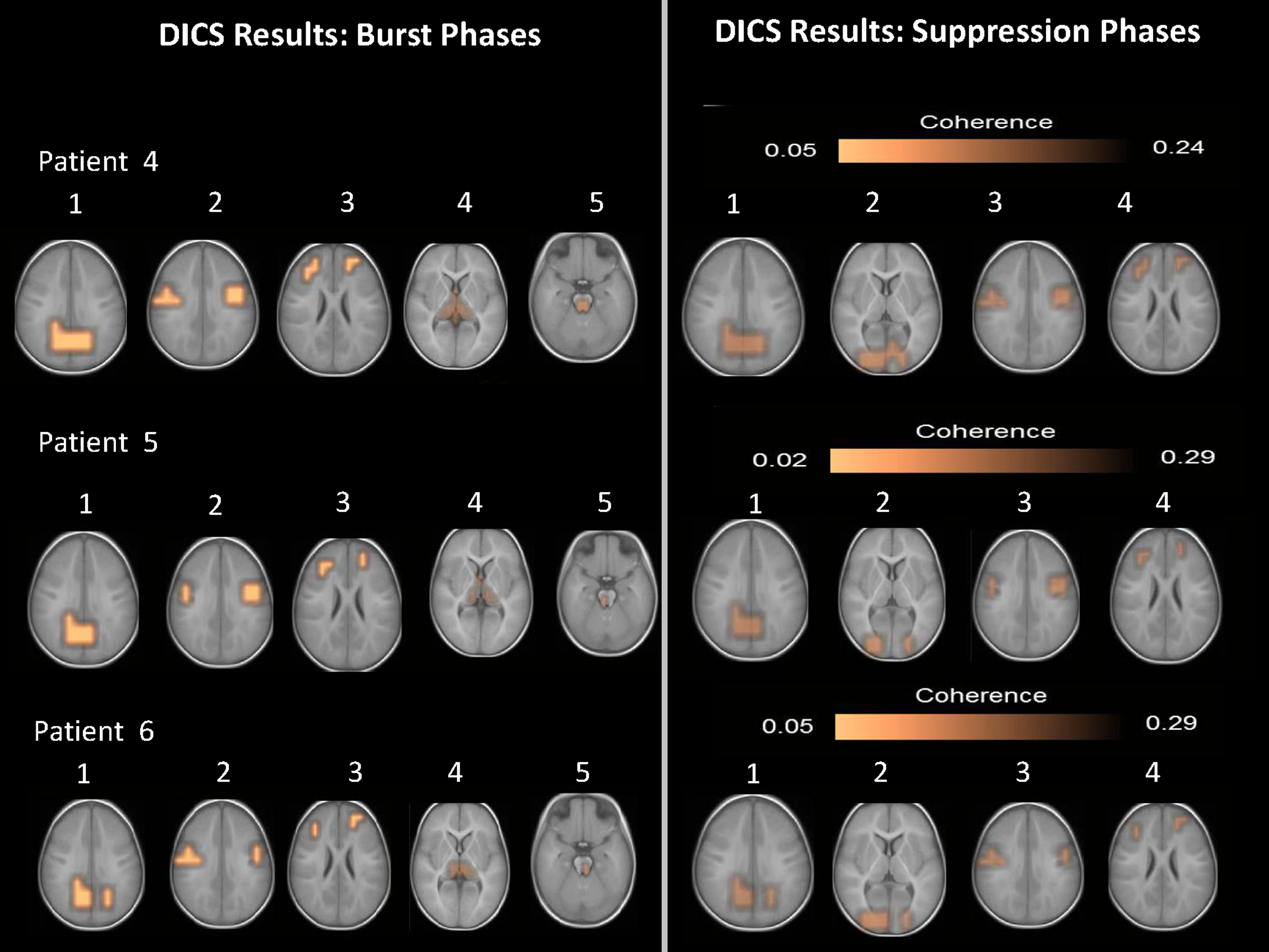

Supplement: S2 Fig — DICS analysis results: showing all sources that were described by DICS in each patient separately for burst and suppression phases, numbered according to the strength of the identified sources. During burst phases: the source of the strongest power (source 1) in the frequency band 1–4 Hz was detected bilaterally in the precuneus in all 13 patients. The local maximum of this source varied across the patients. In all the cases, there were four sources coherent with the first source. Sources with the strongest coherence with the reference (source 2) source were found bilaterally in the somatosensory cortex in the patients. The next strongest source (source 3) were detected in prefrontal regions bilaterally; subsequent sources were found in the thalamus (source 4) bilaterally in eleven patients and unilaterally on the left side in two patients, whereas the last coherent source was determined in the brainstem (source 5), or more precisely in the mid-brain tegmentum in all thirteen patients. During suppression phases: The source of the strongest power (source 1) in the frequency band 1–4 Hz was detected bilaterally in the precuneus in all 13 patients. The second strongest sources were found bilaterally in the occipital cortex (source 2) in eleven patients and unilaterally on the left side in two patients. The next strongest coherence was detected bilaterally in the somatosensory cortex (source 3), the location of these sources were analogous to the second sources during the burst phases. The last coherent sources were determined bilaterally in prefrontal cortex (source 4). (TIF) [file pone.0123807.s002.tif]

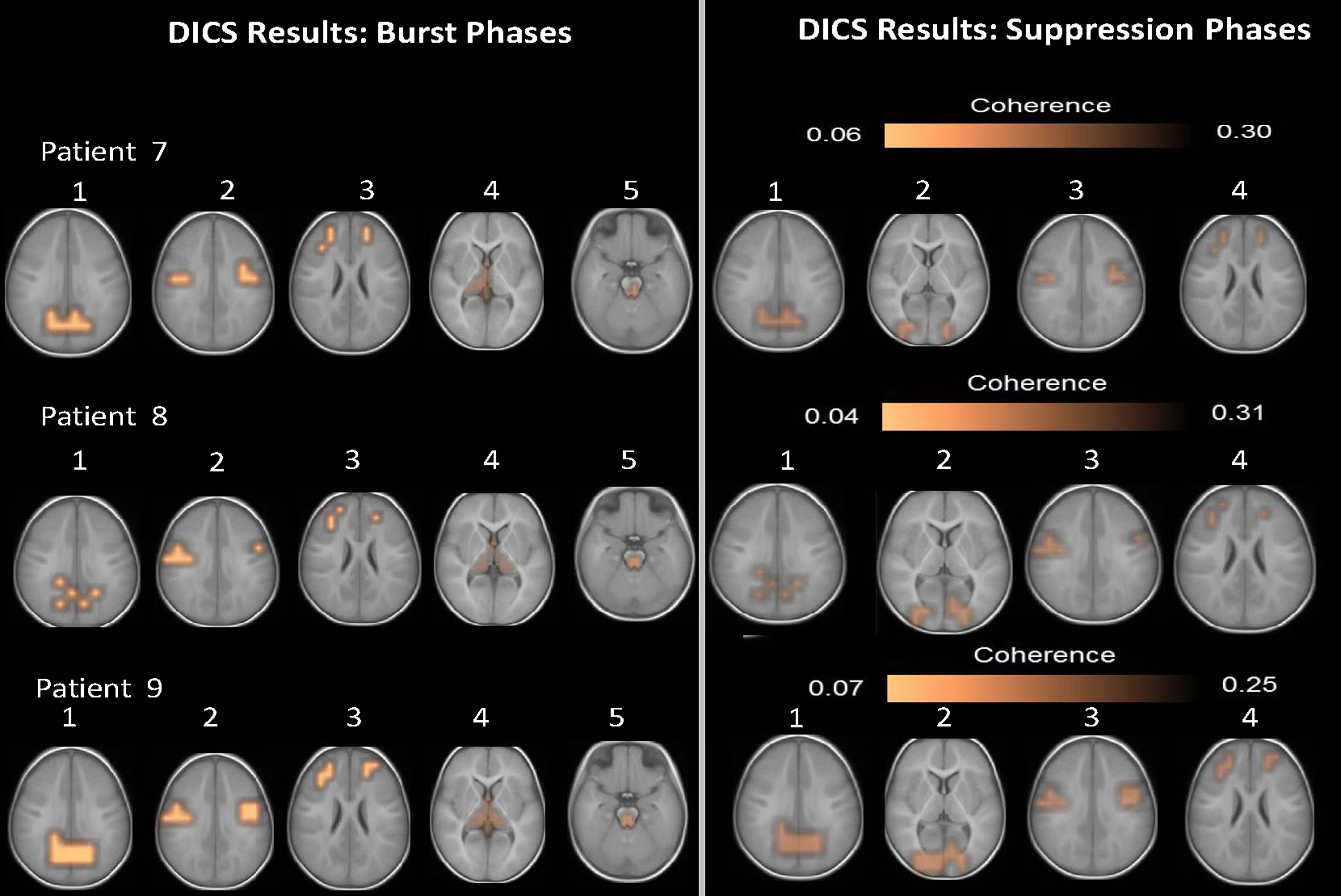

Supplement: S3 Fig — DICS analysis results: showing all sources that were described by DICS in each patient separately for burst and suppression phases, numbered according to the strength of the identified sources. During burst phases: the source of the strongest power (source 1) in the frequency band 1–4 Hz was detected bilaterally in the precuneus in all 13 patients. The local maximum of this source varied across the patients. In all the cases, there were four sources coherent with the first source. Sources with the strongest coherence with the reference (source 2) source were found bilaterally in the somatosensory cortex in the patients. The next strongest source (source 3) were detected in prefrontal regions bilaterally; subsequent sources were found in the thalamus (source 4) bilaterally in eleven patients and unilaterally on the left side in two patients, whereas the last coherent source was determined in the brainstem (source 5), or more precisely in the mid-brain tegmentum in all thirteen patients. During suppression phases: The source of the strongest power (source 1) in the frequency band 1–4 Hz was detected bilaterally in the precuneus in all 13 patients. The second strongest sources were found bilaterally in the occipital cortex (source 2) in eleven patients and unilaterally on the left side in two patients. The next strongest coherence was detected bilaterally in the somatosensory cortex (source 3), the location of these sources were analogous to the second sources during the burst phases. The last coherent sources were determined bilaterally in prefrontal cortex (source 4). (TIF) [file pone.0123807.s003.tif]

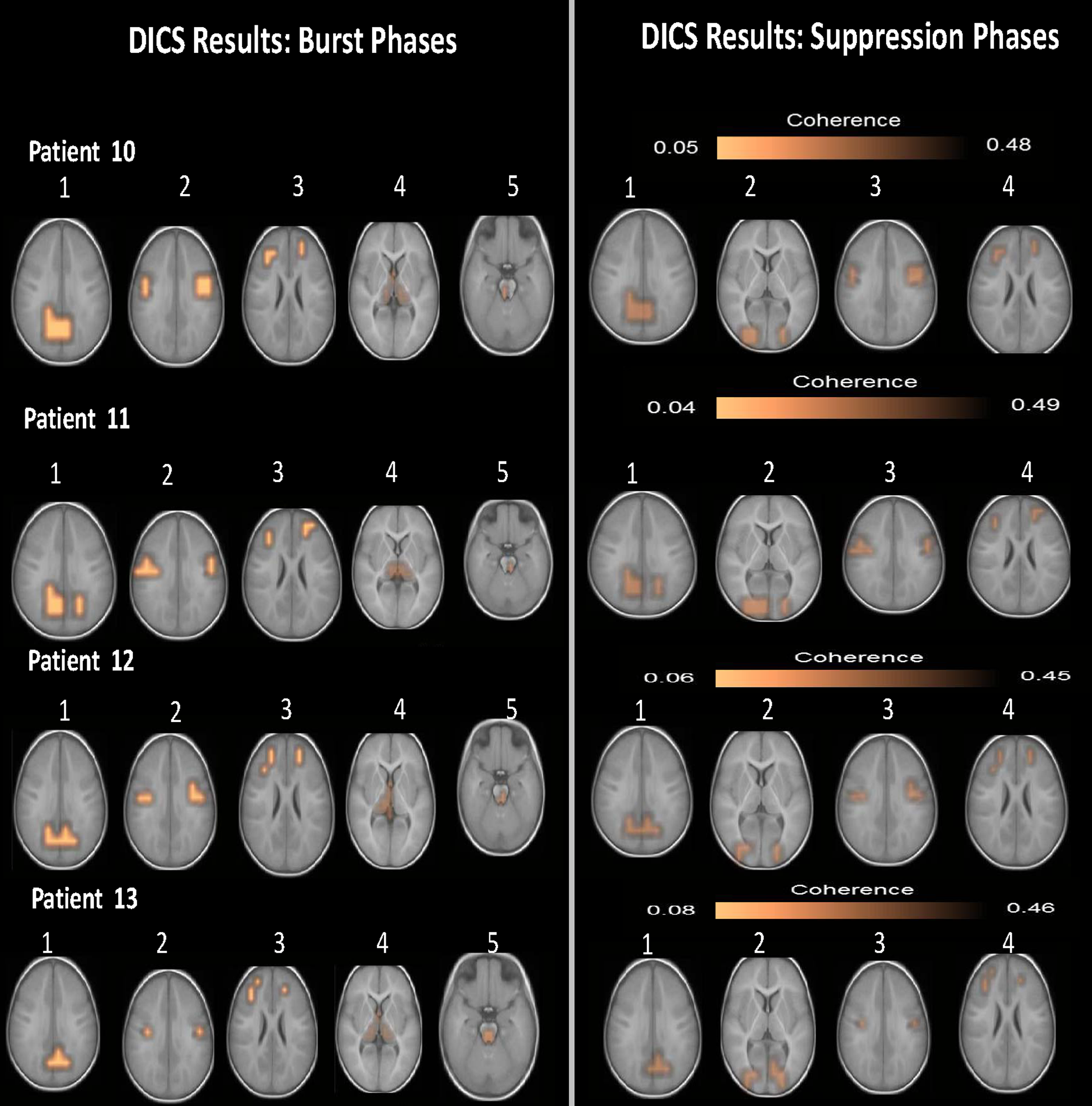

Supplement: S4 Fig — DICS analysis results: showing all sources that were described by DICS in each patient separately for burst and suppression phases, numbered according to the strength of the identified sources. During burst phases: the source of the strongest power (source 1) in the frequency band 1–4 Hz was detected bilaterally in the precuneus in all 13 patients. The local maximum of this source varied across the patients. In all the cases, there were four sources coherent with the first source. Sources with the strongest coherence with the reference (source 2) source were found bilaterally in the somatosensory cortex in the patients. The next strongest source (source 3) were detected in prefrontal regions bilaterally; subsequent sources were found in the thalamus (source 4) bilaterally in eleven patients and unilaterally on the left side in two patients, whereas the last coherent source was determined in the brainstem (source 5), or more precisely in the mid-brain tegmentum in all thirteen patients. During suppression phases: The source of the strongest power (source 1) in the frequency band 1–4 Hz was detected bilaterally in the precuneus in all 13 patients. The second strongest sources were found bilaterally in the occipital cortex (source 2) in eleven patients and unilaterally on the left side in two patients. The next strongest coherence was detected bilaterally in the somatosensory cortex (source 3), the location of these sources were analogous to the second sources during the burst phases. The last coherent sources were determined bilaterally in prefrontal cortex (source 4). (TIF) [file pone.0123807.s004.tif]

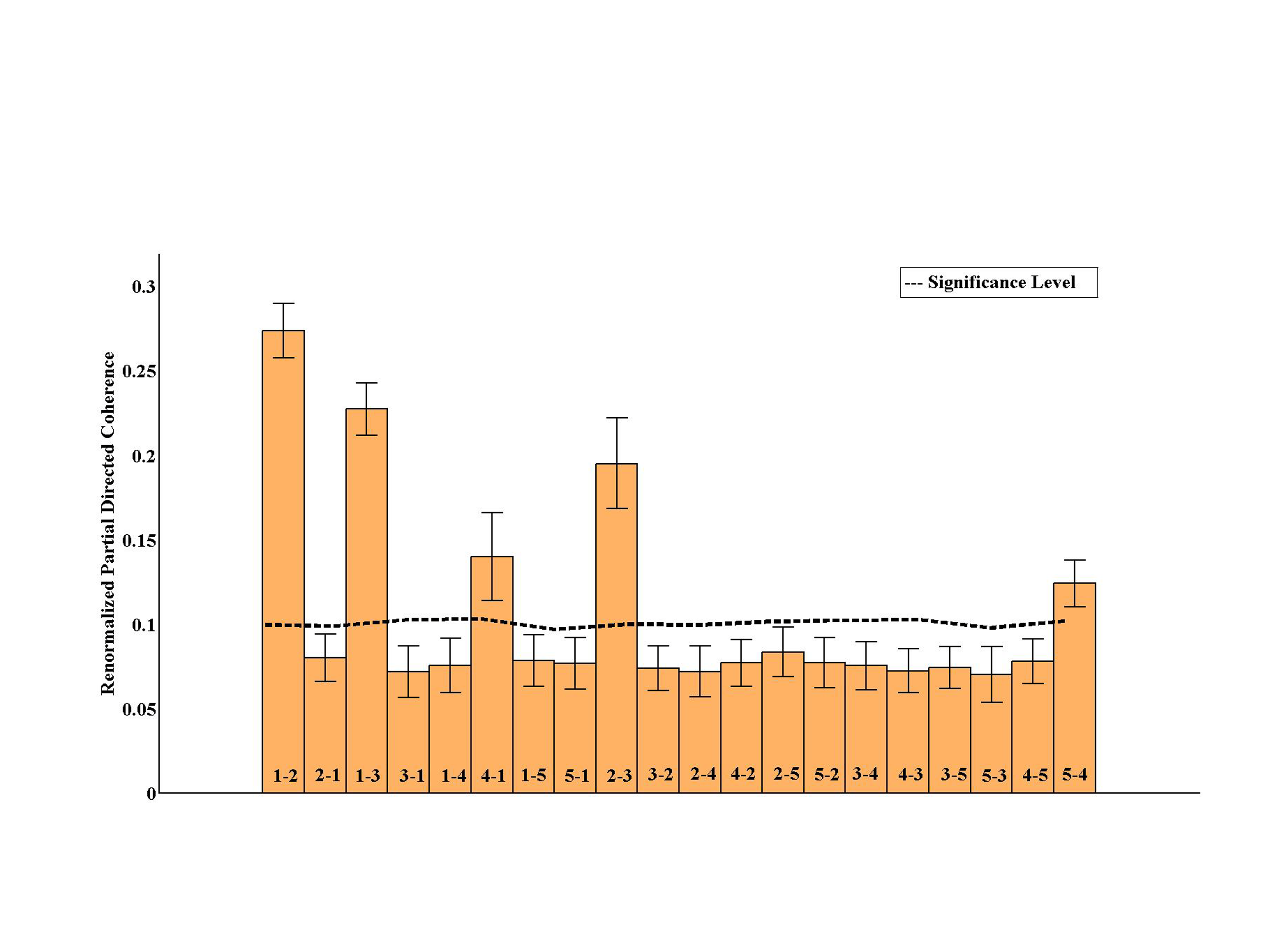

Supplement: S5 Fig — Each bar in the graphs shows the mean and standard deviation of the strength of information flow between two given sources, separately for each direction. Dashed line illustrates the surrogate data driven significance level. (TIF) [file pone.0123807.s005.tif]

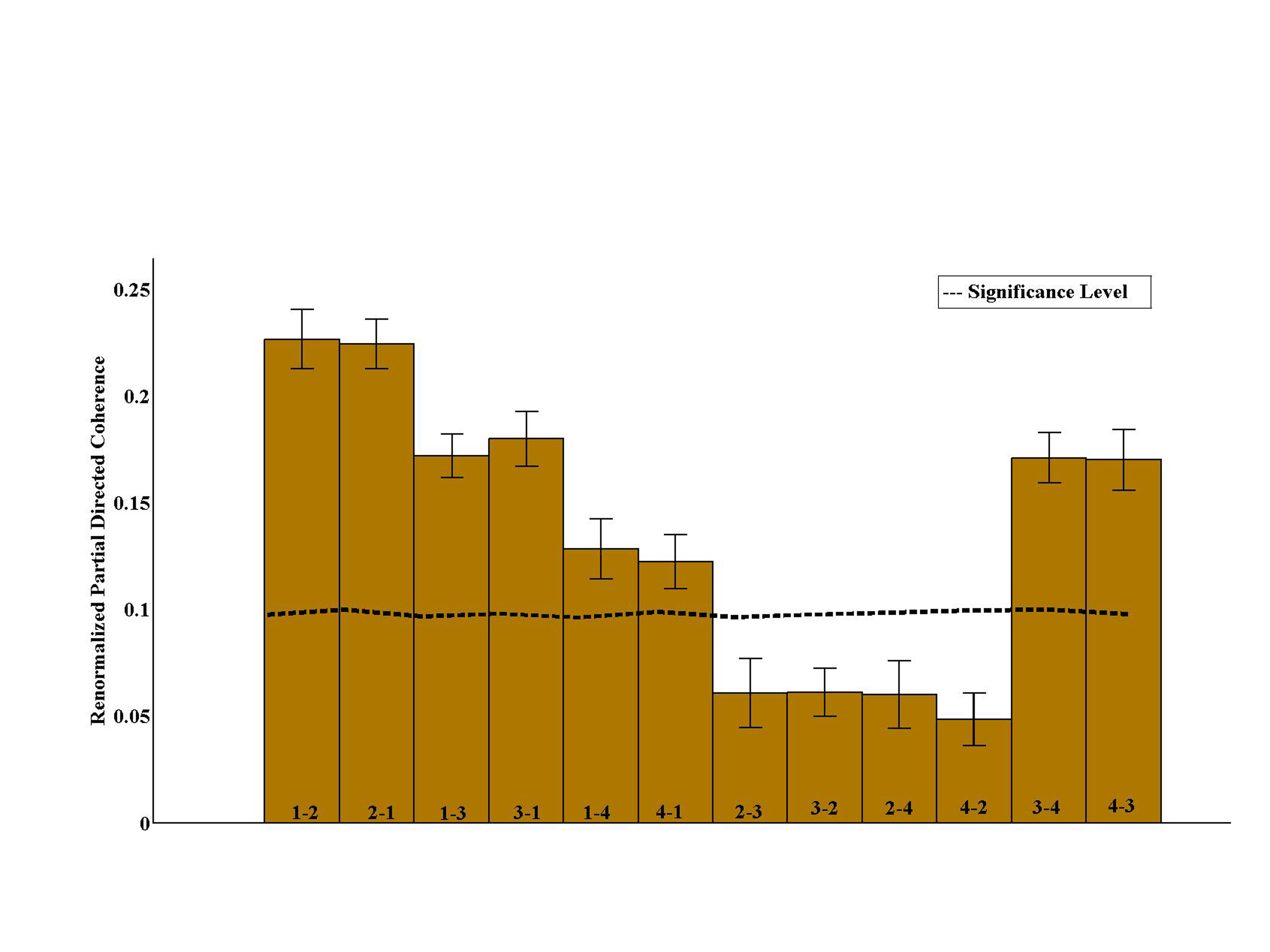

Supplement: S6 Fig — Each bar in the graphs shows the mean and standard deviation of the strength of information flow between two given sources, separately for each direction. Dashed line illustrates the surrogate data driven significance level. (TIF) [file pone.0123807.s006.tif]
